# Prevalence and Associated Factors of Medication Non-Adherence in CRS Patients following Endoscopic Sinus Surgery

**DOI:** 10.3390/jcm12165381

**Published:** 2023-08-18

**Authors:** Shyam Ajay Gokani, Allan Clark, Amin Javer, Carl Philpott

**Affiliations:** 1 Norwich Medical School, University of East Anglia, Norwich NR4 7TJ, UK; s.gokani@uea.ac.uk (S.A.G.); allan.clark@uea.ac.uk (A.C.); 2 St Paul’s Sinus Centre, Vancouver, BC V6Z 1Y6, Canada; sinusdoc@me.com; 3 James Paget University Hospital NHS Foundation Trust, Gorleston-on-Sea, Great Yarmouth NR31 6LA, UK

**Keywords:** chronic rhinosinusitis, endoscopic sinus surgery, medication adherence, paranasal sinus diseases, patient compliance

## Abstract

This study aimed to evaluate factors that may predispose patients to not adhere to prescribed medication after endoscopic sinus surgery (ESS) and to compare SNOT-22 scores at 0–12 months post-operatively between adherent and non-adherent patients. CRS patients who underwent ESS between 2012 and 2016 were recruited to this retrospective cohort study. Adherence was assessed through a questionnaire and review of medical notes. Ninety-four participants were included (61% male, mean age 60). Of those, 66% did not adhere to their prescribed post-operative CRS medication timing or dosage. The most common reason for non-adherence was improvement of symptoms (17%), followed by deterioration of symptoms (11%) and side effects (10%). Post-operative SNOT-22 scores were lowest for non-intentionally non-adherent (NINA) participants with a mean of 10.5 [95% CI: 7.47–13.5], compared to 25.0 for intentionally non-adherent (INA) [95% CI: 17.6–32.4] and 17.7 for adherent patients [95% CI: 13.7–21.7], *p* = 0.01. This study identifies that almost two-thirds of patients are not compliant with CRS medications after ESS. NINA participants reported lower post-operative SNOT-22 scores compared to INA and adherent participants. Future studies should focus on educating patients to continue with medications post-operatively despite an initial improvement in symptoms.

## 1. Introduction

Medication compliance is defined as adherence to a prescribed treatment or following the instructions given by a health care practitioner, in accordance with the prescribed interval, dosage and frequency [1,2,3]. Failure to adhere to recommended treatments results in a considerable burden to health care systems and poor medical outcomes [3,4]. Therefore, adherence is an important factor in the effectiveness of medical therapies and is particularly critical in the treatment of long-term conditions such as chronic rhinosinusitis (CRS) [5,6]. Adherence to intranasal corticosteroids has been shown to be associated with improvement in CRS symptoms such as rhinorrhoea and nasal obstruction [7].

### 1.1. Rationale

One in three patients with CRS are reported to have poorly controlled symptoms in primary care [8], and the annual cost of CRS treatment in the USA is estimated at a total of over $10 billion [9]. Current guidelines recommend endoscopic sinus surgery as the next step for those who fail medical management alone; however, many patients undergo revision surgery after a period of time, and an estimated £15 million is spent annually on revision sinus surgery or nasal polypectomies in the UK [10]. Due to the prevalence and expense of CRS, continued efforts are needed to establish cost-effective strategies for patient management across primary and secondary care [11].

Unfortunately, adherence to treatment regimens in CRS is persistently impaired. Usage of intranasal corticosteroids in CRS is reported to be as low as 18%, and as low as 1% for nasal douching [12]. In developed countries, adherence to other chronic illness regimens is as low as 50% [1,13,14,15] and is associated with poorer clinical outcomes [2]. To improve patient adherence, it is important to recognise any factors that may lead to non-adherence [13].

### 1.2. Objectives

The objectives of this study were to evaluate factors that may predispose patients to non-adherence to prescribed medications after endoscopic sinus surgery (ESS) and to compare SNOT-22 scores 0–12 months post-operatively between adherent and non-adherent patients. 

## 2. Materials and Methods

### 2.1. Study Design

A cohort study was performed to capture retrospective data on included participants. All subjects provided written informed consent. There was no reimbursement or incentive for participation in this study.

### 2.2. Participants and Setting

Adult patients over 18 years of age who underwent ESS for CRS at James Paget University Hospital between 2012 and 2016 were considered for inclusion if they met the CRS diagnosis criteria outlined in the European Position Paper on Rhinosinusitis and Nasal Polyps (EPOS 2012). Participants all had a >12-week history of nasal congestion and/or nasal discharge along with hyposmia and/or facial pressure/pain and confirmation of disease on endoscopy and/or CT scan. All participants consented to undergo ESS as part of their treatment pathway.

Exclusion criteria were as follows:

- Rare or complex sinus conditions
- CRS secondary to systemic disease such as cystic fibrosis and granulomatous disease
- Suspected malignancy
- Pregnant or lactating women
- Immunodeficiency states including HIV and selective or multiple antibody deficiency states
- Inability to give consent or to understand and comply with study instructions

### 2.3. Variables

Data extracted included age, gender, ethnicity, aspirin and non-steroidal anti-inflammatory drug (NSAID) sensitivity status, pre-operative Lund-Mackay CT scores and pre- and post-operative SNOT-22 scores (at 3, 6, 12 and >12 months following ESS). A questionnaire for participants was created to assess adherence to prescribed treatment and to assess whether patients noted a positive or negative impact of these treatments on their health. All patients were asked the same 25 questions regarding their demographic characteristics, smoking and allergy status, family and CRS history, comorbidities and type of treatment prescribed, including their perceptions of treatment and adherence (Supplementary Materials). Questionnaires were administered 12 months following surgery or at the time of last follow-up, whichever was later. 

### 2.4. Data Sources

Data were extracted from the hospital electronic medical record system and entered into a standardised collection form. All clinical notes for relevant visits were reviewed. Any missing data from the questionnaire were completed in accordance with participants’ medical notes. If treatment information was not available it was defined as not taken or not useful. 

### 2.5. Methods Used to Reduce Potential Bias

All eligible patients that attended our institution for ESS between 2012 and 2016 were approached twice for inclusion, independent of age, sex, ethnicity or post-surgical results. After this, they were considered non-responders. All responses were verified objectively against the patient’s medical records, to improve the accuracy of data on medication usage and adherence as far as possible. 

### 2.6. Sample Size

Cochran’s equation was used to estimate the sample size [16]. For this study, a case-scenario of an adherence rate of 50% was assumed as the worst-case scenario. A sample size of 100 participants would allow for the estimation of adherence with a 10% margin (range, 40–60%) using a 95% confidence interval (CI).

### 2.7. Quantitative Variables

The study population was divided into three different groups according to their reported adherence with prescribed medication: adherent, non-intentionally non-adherent (NINA) and intentionally non-adherent (INA). INAs were defined as participants who reported stopping their CRS medication due to side effects, worsening symptoms or improvement of symptoms, and who were also identified as non-adherent from their medical records. This was confirmed as participants who answered ‘yes’ to questionnaire items 21, 22 or 23. NINAs were defined as participants who reported forgetting to take their CRS medication or who reported not always taking their medication at the correct time, and who were also identified as non-adherent from their medical records. This was confirmed as participants who answered ‘yes’ to questionnaire item 19 or ‘no’ to item 20. Adherence was at 12 months following surgery or at the time of last follow-up, whichever was later.

### 2.8. Statistical Methods

Statistical analysis was performed using RStudio (version 1.1.383, RStudio, Inc., Boston, MA, USA). For continuous variables, results were expressed as means and standard deviations or in box plots. Data distribution was tested for normality using the Shapiro–Wilk test. For dichotomous variables, frequencies and percentages were calculated. For continuous variables, we compared the means between adherent and non-adherent groups using a Student’s *t*-test where the distribution was normal; otherwise, we used a Mann–Whitney test. In univariate analyses, categorical variables were compared between adherent and non-adherent using chi-squared tests. Adherence was estimated as the percentage of patients who were fully adherent to the drug regime prescribed. The 95% CIs were estimated to present uncertainty in the estimate. The distribution of demographic and clinical variables was examined among the different adherent groups. A *p*-value less than 0.05 was considered statistically significant.

## 3. Results

### 3.1. Participants and Descriptive Data

A total of 158 patients who underwent ESS during the study period were approached. Of these, 94 participants (59.5%) chose to participate in the study. The characteristics of our study population are shown in Table 1. A total of 57 male and 37 female participants were included with a mean age of 60 years. Over 70% of participants were also taking other medications for a non-rhinological disease, such as antihypertensives. A total of 45 patients (48%) had comorbid asthma.

### 3.2. Outcome Data

#### 3.2.1. Prevalence of Medication Adherence

**Figure 1:**
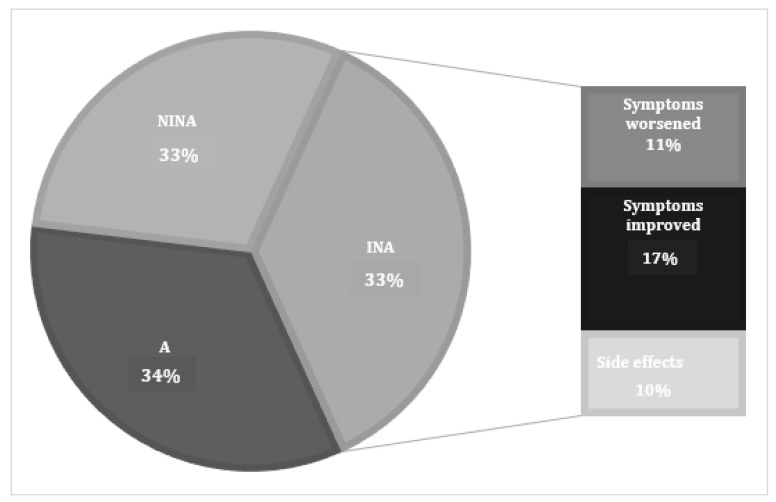
Patient self-reported adherence to medications for CRS. Key: A—adherent; INA—intentionally non-adherent; NINA—non-intentionally non-adherent.

**Figure 2:**
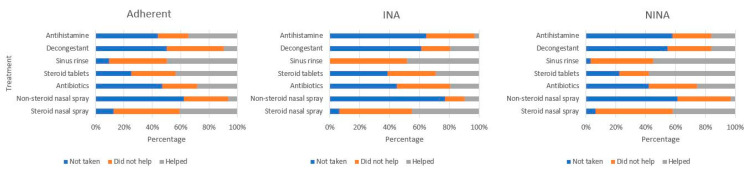
Patient usage and perception of help for CRS medications.

Full adherence to their drug regimen was reported by 32 patients (34%). 31 participants (33%) unintentionally did not adhere to their prescribed treatment time or dosage (non-intentional non-adherents—NINAs) and 31 participants (33%) stopped medical therapy deliberately (intentional non-adherents—INAs). The most common reason for INA was improvement of symptoms, followed by deterioration of symptoms despite medication usage and then side effects from medication (Figure 1). Figure 2 shows patient usage by type of medication prescribed in all participants, characterised by adherence status. Steroid tablets and sinus rinses were reported as the most helpful medical therapies, while non-steroidal sprays, antibiotics and nasal decongestants were reported as the least helpful of all treatments.

#### 3.2.2. Predictors of Adherence

The association of demographic and clinical characteristics of participants to adherence status is shown in Table 1. There was no difference in age, duration of CRS symptoms, and preoperative Lund–Mackay between adherent patients, INAs and NINAs. Pre-operative SNOT-22 scores also did not differ between groups with mean differences in INA versus adherent patients of 10.93 (−2.89, 24.74), NINA versus adherent of 4.81 (−9.90, 19.53) and NINA versus INA of −6.11 (−20.96, 8.73).

#### 3.2.3. Post-Operative SNOT-22 Scores

**Figure 3:**
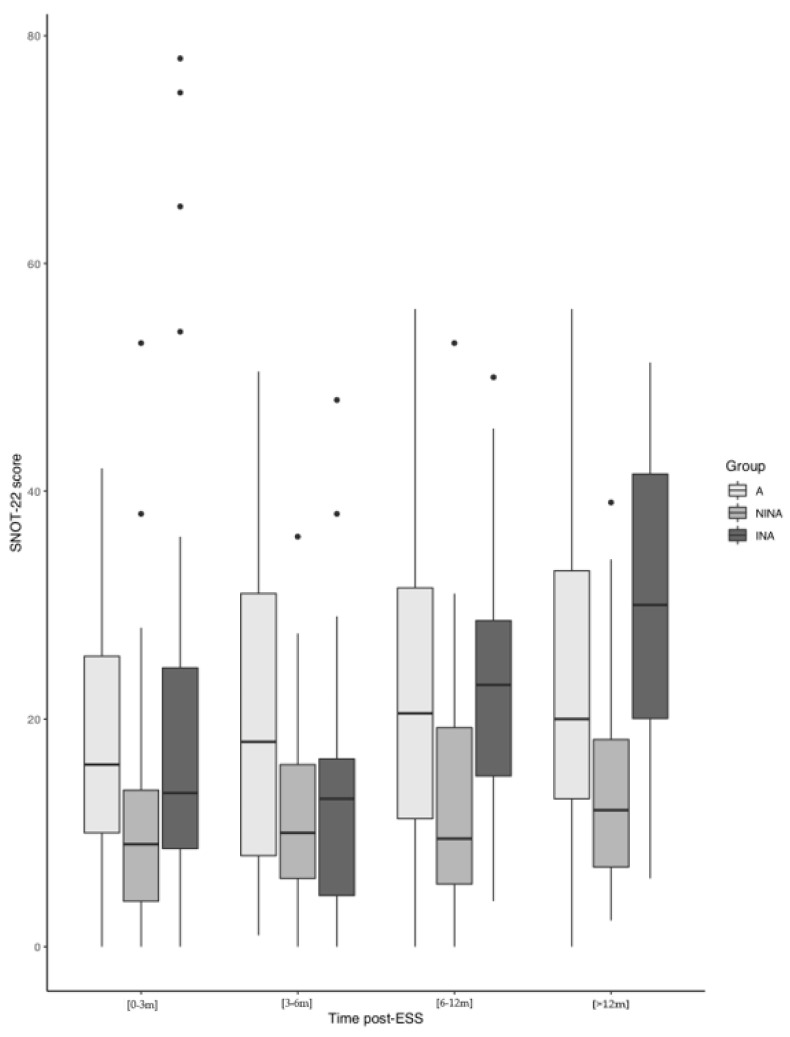
Evolution of SNOT-22 scores over the post-operative period according to adherence.

Post-operative SNOT-22 scores were lowest for NINAs, with a mean difference of −14.52 (95% CI: −23.48–−5.56) compared to INA, and −7.17 (95% CI: −15.96–1.63) compared to adherent patients. Overall, post-operative SNOT-22 scores improved at 12 months in all groups but this difference was not statistically significant (Figure 3). NINAs had comparatively lower mean SNOT-22 scores that slowly increased after one year of surgery, whilst INAs experienced a greater deterioration in SNOT-22 scores after surgery. Mean SNOT-22 scores for adherent patients did not vary significantly over time. 

## 4. Discussion

### 4.1. Key Results

Adherence to medical treatment post-operatively is an important component of CRS patient management following ESS. Almost two-thirds of patients in this study were not adherent to medications, suggesting that post-surgical adherence in patients with CRS is a target for improvement in clinical practice. In order to do so, we must understand and address any factors that drive this poor adherence.

In our study, better quality of life outcomes after ESS were achieved by patients with non-intentionally non-adherent behaviour. This may be attributable to patients forgetting to take their treatment due to a low symptom burden, resulting in fewer prompts and reduced motivation to adhere to treatment. Our study assessed adherence at a minimum of 12 months post-operatively. A longer follow-up duration may have revealed a subsequent deterioration in SNOT-22 scores for non-adherent patients, so future studies should explore the difference in outcomes between adherent and non-adherent patients beyond 12 months post-operatively. Wahid et al. quantified medication usage by measuring direct medication costs in a case-control study of 139 CRS patients. They found an inverse relationship between medication usage and health-related quality of life, supporting the paradoxical relationship between adherence and SNOT-22 scores seen in our study [17].

The most common reason in this study for intentional non-adherence was symptom improvement. This highlights the need to counsel CRS patients about their disease course and emphasise that non-adherence to treatment may lead to recurrence of symptoms later on, despite initial post-operative improvement in quality of life. 

### 4.2. Limitations

This was a single-centre study, which may not provide a representative sample of the wider CRS population. For example, the average age for participants in this study was 60 years, which was higher than in other studies of CRS patients [7,17,18,19]. This could be due to the local demographics of the study site or the fact that older participants may have been more likely to respond to the survey. However, we did not observe any differences in outcomes associated with participant age. Although the questionnaires were simple to administer, self-reported adherence may not reflect true clinical practice and there was a potential for recall bias due to the 12-month lag between surgery and questionnaire completion for participants. However, in order to mitigate this, we correlated questionnaire responses with clinical notes and observed an under-reporting of adherence in 5% of the responders when comparing self-reported and actual medication adherence. This may ultimately lead to an overestimation of adherence levels. We defined participants as either adherent or non-adherent but did not quantify the level of adherence, as this was difficult to verify through clinical notes. Further insights may have been possible by categorising patients by level of adherence. Finally, approximately 40% of patients did not respond to this survey. Since non-responders may have been more likely to be non-adherent to medications, this may have also led to an overestimation of adherence within our cohort.

### 4.3. Interpretation of Findings and Generalisability

Although multiple studies have explored adherence in asthma, COPD and other chronic respiratory illnesses [20,21,22,23], there are limited prospective data on adherence in post-operative CRS patients [24,25,26,27,28,29,30,31]. Qualitative and case-control studies have demonstrated that patients are frequently non-compliant with medications [23,28]. At the point of referral to secondary care, less than 20% use intranasal steroids and less than 2% perform nasal douching [12], despite national guidelines to the contrary [32,33]. 

Previous studies have found lower age to be associated with non-adherence to post-ESS medication [20,34,35,36,37,38]. This relationship was not observed in our study, perhaps due to the older demographic of our population. Previous studies have also demonstrated an association between education levels and medication adherence in other chronic disease states [13,36,38,39] but this area is underexplored in the context of CRS and would be an interesting area of focus for future work. Phillips et al. found in a study of 174 CRS patients followed for 12 months that adherence with intranasal corticosteroids was associated with comorbid asthma and allergy and that intranasal steroids had better adherence than saline irrigations [5]. Although we did not observe such a relationship in our study, saline rinses were reported as more useful than intranasal steroids.

Similarly, Nabi et al. found that pre-operative SNOT-22 scores predicted adherence to nasal sprays 12 months after ESS in a study of 60 patients [27]. They found 57.4% of patients to be non-adherent, which is similar to our finding of 66% non-adherence. In our study, pre-operative SNOT-22 scores did not differ between groups so we could not explore this relationship; instead, we found post-operative SNOT-22 scores to be more predictive. Given that the primary reason for intentional non-adherence in our study was symptom improvement, measures to educate patients on the need for continuing treatment should be explored. Feng et al. investigated the use of a social media messaging app (WeChat) in China to encourage the use of intranasal corticosteroids after ESS. They found that patients randomised to receive daily WeChat prompts had higher adherence at three months compared to controls, suggesting that digital tools are a potential area of focus to educate patients. Furthermore, in our centre, further work is underway to identify biomarker profiles which may help to predict patient outcomes following medical and surgical treatment, enabling a personalised approach to treatment. The optimal patient cohort who is most likely to benefit from long-term sinus care in the form of saline nasal rinses and topical steroids is still unknown. NINA participants in our study may be appropriately de-escalating care. Personalising treatment to individual patient biology may enable improved adherence, as patients are more likely to be prescribed appropriate treatment regimens. 

Studies of patients suffering from other chronic diseases such as hypertension have identified that intentional non-adherence is driven by patient beliefs about their illness or treatment, whilst unintentional non-adherence is more closely related to demographic factors such as age [40]. Understanding the relationship between intentional and unintentional non-adherence is important to developing targeted strategies to improve adherence [41,42].

### 4.4. Future Work

Future prospective studies are required to further investigate predictors of medication adherence post-ESS. Multi-centre studies with follow-up beyond 12 months, using standardised measures such as the SNOT-22 score, are essential to explore the relationship between adherence and post-operative quality of life. The ongoing MACRO randomised controlled trial aims to follow participants for up to five years after interventions that include endoscopic sinus surgery [31]. This will further qualify the counter-intuitive relationship between NINA and improved SNOT-22 scores seen in our study. Additional predictors such as education level and socioeconomic status are also important to study. The use of digital tools to promote patient education and endotyping to improve treatment selection are emerging areas of research for improved adherence. Future studies should aim to quantify the level of adherence to medications and the impact of this on clinical outcomes. Furthermore, future data on surgical complication rates and the association with adherence would be useful to explore through multivariate analyses in larger patient cohorts. 

The advent of biologic drugs in the United Kingdom for CRS patients with severe comorbid asthma warrants an improved understanding of the factors which impair medication adherence. Although the use of biologic drugs for CRS is currently limited to a small number of patients, candidates for biologic therapy may need to demonstrate adherence to courses of oral corticosteroids [43]. Some patients who are least able to comply with standard treatments for CRS may benefit from treatment with biologic agents. Future studies should, therefore, focus on the role of biologic drugs in non-adherent CRS patients.

## 5. Conclusions

This study identifies that almost two-thirds of patients are not compliant with CRS medications after endoscopic sinus surgery, with symptom improvement being the most common reason for INA. NINA participants reported lower post-operative SNOT-22 scores compared to INA and adherent participants. Future studies should focus on exploring quality of life outcomes beyond 12 months post-surgery and on educating patients to continue with medications post-operatively despite an initial improvement in symptoms. 

## Figures and Tables

**Table 1:** Demographic and clinical characteristics of included patients categorised by self-reported adherence to CRS medications.

Characteristic		A	INA	NINA	*p*-Value
N		32	31	31	
Gender	Female	14 (44%)	10 (32%)	13 (42%)	0.61
	Male	18 (56%)	21 (68%)	18 (58%)	
Ethnicity	British	30 (94%)	28 (90%)	30 (97%)	0.34
	Caribbean	0 (0%)	1 (3%)	0 (0%)	
	Irish	2 (6%)	0 (0%)	0 (0%)	
	Other	0 (0%)	1 (3%)	1 (3%)	
	White and black African	0 (0%)	1 (3%)	0 (0%)	
Age, mean (SD)		61.03 (11.72)	58.84 (7.93)	59.03 (9.80)	0.63
Smoking status	Ex-smoker	17 (53%)	10 (32%)	13 (42%)	0.55
	Non-smoker	14 (44%)	19 (61%)	17 (55%)	
	Smoker	1 (3%)	2 (6%)	1 (3%)	
Time suffering from CRS in years, mean (SD)		21.83 (13.92)	22.50 (11.40)	21.98 (13.99)	0.98
SNOT-22 preop, mean (SD)		38.24 (24.64)	49.17 (23.96)	43.05 (23.94)	0.29
SNOT-22 post op, mean (SD)		17.69 (11.48)	25.04 (21.43)	10.52 (8.65)	0.008
LM score, mean (SD)		18.41 (5.402)	15.83 (7.33)	19.04 (5.04)	0.14
Presence of comorbidities	No	6 (19%)	11 (35%)	9 (29%)	0.43
	Yes	26 (81%)	20 (65%)	22 (71%)	
Family history of airway disorders	No	26 (81%)	20 (64%)	21 (67%)	0.28
	Yes	6 (19%)	11 (35%)	10 (32%)	
Presence of nasal allergies	No	15 (47%)	11 (35%)	13 (42%)	0.66
	Yes	17 (53%)	20 (65%)	18 (58%)	
Presence of NSAID allergy	No	25 (78%)	27 (87%)	23 (75%)	0.53
	Yes	7 (22%)	4 (13%)	8 (26%)	
Asthma	No	11 (34%)	18 (58%)	19 (61%)	0.065
	Yes	21 (66%)	13 (42%)	12 (39%)	

Key: A—adherent; INA—intentionally non-adherents; NINA—non-intentionally non-adherents. SNOT-22 preop—preoperative sinonasal outcome test-22 score. SNOT-22 postop—postoperative sinonasal outcome test-22 score. LM score—pre-operative Lund Mackay computed tomography score. NSAID—non-steroidal anti-inflammatory drug. CRS—chronic rhinosinusitis.

## Data Availability

The data presented in this study are available on request from the corresponding author.

## Supplementary Materials

The following supporting information can be downloaded at: https://www.mdpi.com/article/10.3390/jcm12165381/s1, Questionnaire.
