# Supplementary material for: Prevalence and Associated Factors of Medication Non-Adherence in CRS Patients following Endoscopic Sinus Surgery
Source: J Clin Med. 2023 Aug 18;12(16):5381. doi: 10.3390/jcm12165381 (PMC10455742; doi:10.3390/jcm12165381)
Supplement: Supplementary file 1 [file jcm-12-05381-s001.zip › jcm-2501562-supplementary.pdf]

Exploring Endotypes in CRS

1. Welcome to the 'Exploring Endotypes in CRS' Study

Welcome to the 'Exploring Endotypes in CRS' Study. Please read the following information carefully before completing the questionnaire. You will have been provided with a Participant Identification Number (PIN) by the study team when you agreed to take part in the study. You will need this PIN number in order to complete the questionnaire. You have been invited to take part because you are either a patient with Chronic Rhinosinusitis or a control subject. If you have volunteered to take part in the study because you are undergoing septoplasty (control subject), not all questions will be relevant to you but please answer the questions wherever possible. If you have previously donated tissue when you had sinus surgery at the James Paget University Hospital, please answer the questions in relation to how you were at the time of coming in for surgery. If you experience any difficulties with this questionnaire or you have any queries related to the study or do not know your PIN number, please contact Jane.Woods@jpaget.nhs.uk.

2. Participant Details

1. Please tell us if you have been invited to complete this questionnaire as a patient with Chronic Rhinosinusitis or as a Control Subject:

- ☐ Patient with Chronic Rhinosinusitis
- ☐ Control Subject

2. Please enter your Participant Information Number (PIN number) - this will have been provided to you by the study team:

3. Please enter your date of birth:

DD/MM/YYYY

4. Please select your gender:

- ☐ Male
- ☐ Female

5. Please select your ethnicity:

- **White**

- ☐ British
- ☐ Irish
- ☐ Other

- **Asian or Asian British**

- ☐ Indian
- ☐ Pakistani
- ☐ Bangladeshi
- ☐ Any other Asian background

- **Mixed**

- ☐ White and Black Caribbean
- ☐ White and black African
- ☐ White and Asian
- ☐ Any other mixed background

- **Black or Black British**

- ☐ Caribbean
- ☐ African
- ☐ Any other black background

- **Other Ethnic Group**

- ☐ Chinese
- ☐ Any other Ethnic Group
- ☐ I do not wish to disclose my ethnic origin

3. Allergies and Smoking History

6. Do you have any allergies?

- ☐ House Dust Mite
- ☐ Timothy Grass

- [ ] Dog
- [ ] Cat
- [ ] Tree Pollen
- [ ] Weed Pollen
- [ ] Aspergillus
- [ ] Alternaria
- [ ] Mixed Moulds
- [ ] Other (please specify):

7. What is your smoking status?

- [ ] Current Smoker
- [ ] Ex-Smoker
- [ ] Non-Smoker

8. If you are a current smoker, how much do you smoke in an average week?

9. If you are an ex-smoker, how long ago did you stop smoking?

Years

Months

4. Medical and Family History

10. Do you have any other medical conditions (for which you receive medical help or take medication)?

11. Is there any family history of medical problems which affect the nose/lungs/sinuses?

5. History of Chronic Rhinosinusitis

12. For how long have you been experiencing symptoms of Chronic Rhinosinusitis (Blocked nose, runny nose, poor sense of smell, facial pressure)?

Years ☐
Months ☐

13. Have you ever received medication for Chronic Rhinosinusitis?

- ☐ Yes
- ☐ No
- ☐ I don't know/I'm not sure

14. Which of the following medications have you received for Chronic Rhinosinusitis?

	Currently taking:	Have taken in the past:	Helped my symptoms:	Did not help my symptoms:
Steroid Nasal Spray	☐	☐	☐	☐
Non-Steroid Nasal Spray	☐	☐	☐	☐
Antibiotics	☐	☐	☐	☐
Steroid Tablets	☐	☐	☐	☐
Sinus Rinse	☐	☐	☐	☐
Nasal Decongestant	☐	☐	☐	☐
Antihistamine	☐	☐	☐	☐
Analgesics (Pain relief such as Paracetamol or similar)	☐	☐	☐	☐

15. If you have taken any of the following medications in the past for Chronic Rhinosinusitis, how many courses have you taken? (Please leave blank if zero).

	Number of Courses/Spells
Steroid Nasal Spray	☐
Non-Steroid Nasal Spray	☐

	Number of Courses/Spells
Antibiotics	☐
Steroid Tablets	☐
Sinus Rinse	☐
Nasal Decongestants	☐
Antihistamines	☐
Analgesics (such as paracetamol or similar)	☐

16. Have you experienced any side effects from current or previous treatments?

17. Have you experienced periods of time where your symptoms of Chronic Rhinosinusitis were well controlled?

- ☐ Yes
- ☐ No
- ☐ I don't know/I'm not sure

18. If you answered yes above, why do you think it was that your symptoms of Chronic Rhinosinusitis were well controlled during this period?

19. Have you ever forgotten to take your medication?

- ☐ Yes
- ☐ No

20. Do you always take your medication at the time indicated?

- ☐ Yes

- [ ] No

21. Do you ever stop taking your medication because your symptoms are worse?

- [ ] Yes
- [ ] No

22. Do you stop taking your medication because your symptoms are better?

- [ ] Yes
- [ ] No

23. Do you ever stop taking medication because of side effects?

- [ ] Yes
- [ ] No

24. In the last week how many times have you not taken your medication?

[ ]
